# Splenic Metastasis in Ewing Sarcoma

**DOI:** 10.5334/jbsr.4203

**Published:** 2026-02-05

**Authors:** Lucas Bijnens, Brecht Van Berkel, Maarten Steyvers

**Affiliations:** 1 Department of Imaging and Pathology, KU Leuven, Leuven, Belgium; 2 Jessa Hospital, Hasselt, Belgium

**Keywords:** Ewing sarcoma, bone tumour, spine, extrapulmonary metastasis, spleen

## Abstract

Ewing sarcoma (ES) is a high-grade osseous malignancy that typically occurs between 10 and 20 years of age. Prognosis is poor with a 30% five-year survival in the case of metastatic disease. This case describes a 20-year-old female patient with known lumbar vertebral ES developing a splenic metastasis years after primary treatment. To the best of our knowledge, metastatic spread to the spleen in ES has not been reported before.

*Teaching point:* In patients with a history of Ewing sarcoma, new parenchymal lesions should always prompt consideration of rare metastatic disease, given its profound prognostic impact.

## Introduction

Ewing sarcoma (ES) is a malignant small blue round cell tumour typically affecting children and young adults. It is associated with a poor prognosis, and only a few treatment options are available [1]. Prognosis is significantly negatively impacted by the presence of distant metastases [2].

ES typically presents as a primary bone tumour, but primary extra-skeletal presentation (e.g. chest wall, pancreas, adrenal gland) is possible [1, 3, 4]. Metastatic spread most frequently targets lungs and bones, but rare parenchymatous abdominal metastases (e.g. pancreas, small bowel) have been reported. To date, metastatic involvement of the spleen has not been reported [1, 5].

Splenic lesions have a broad differential diagnosis and can pose a diagnostic challenge [6, 7]. Since the presence of metastatic disease highly influences the prognosis of ES, it is important to be aware of possible splenic involvement.

## Case Report

**Figure 1:**
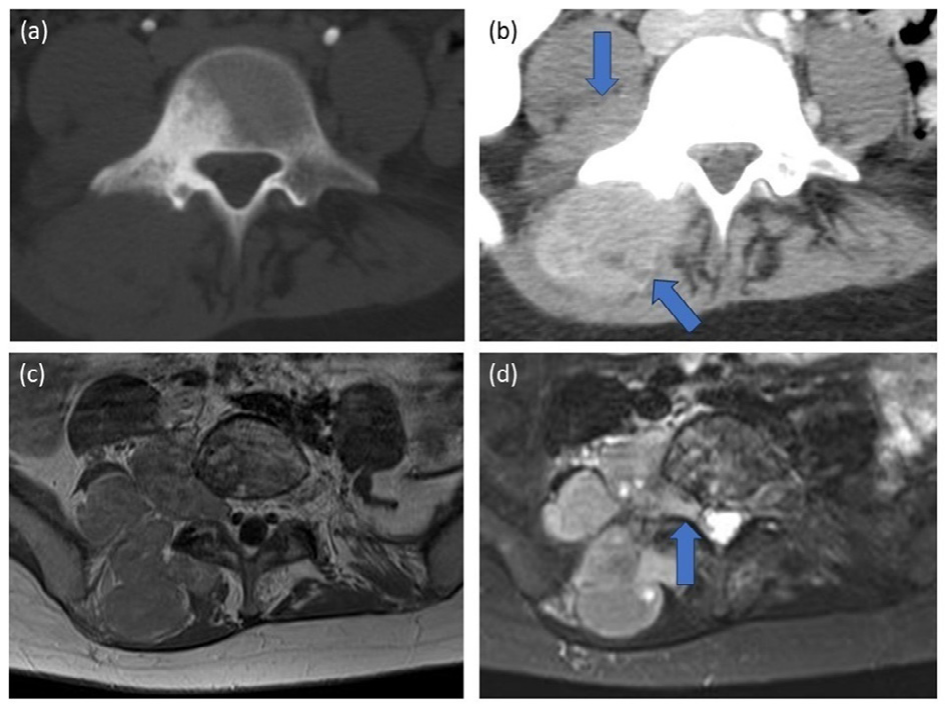
Primary diagnosis. **(a, b)** Abdominal CT. An irregular sclerotic lesion in hemicorpus L5, with extensive associated paravertebral soft tissue involvement (5.5 × 6.5 × 6.0 cm) (arrows). **(c, d)** Spinal MRI. A T2-hyperintense, heterogeneously contrast-enhancing mass in hemicorpus L5, with associated soft tissue involvement extending into the intervertebral foramen L4-L5 (arrow).

At the age of 20, our patient was diagnosed with ES in the right hemicorpus of L5, extending into the paravertebral soft tissues (L4-S1) (Figure 1). A CT-guided biopsy confirmed the diagnosis of ES. A millimetric osseous lesion with similar morphology was present in the right iliac wing at the time of diagnosis and considered a probable metastasis. Further work-up did not reveal any other metastases. The patient showed a complete response upon chemotherapy treatment, without signs of recurrence or distant metastases during regular follow-up.

**Figure 2:**
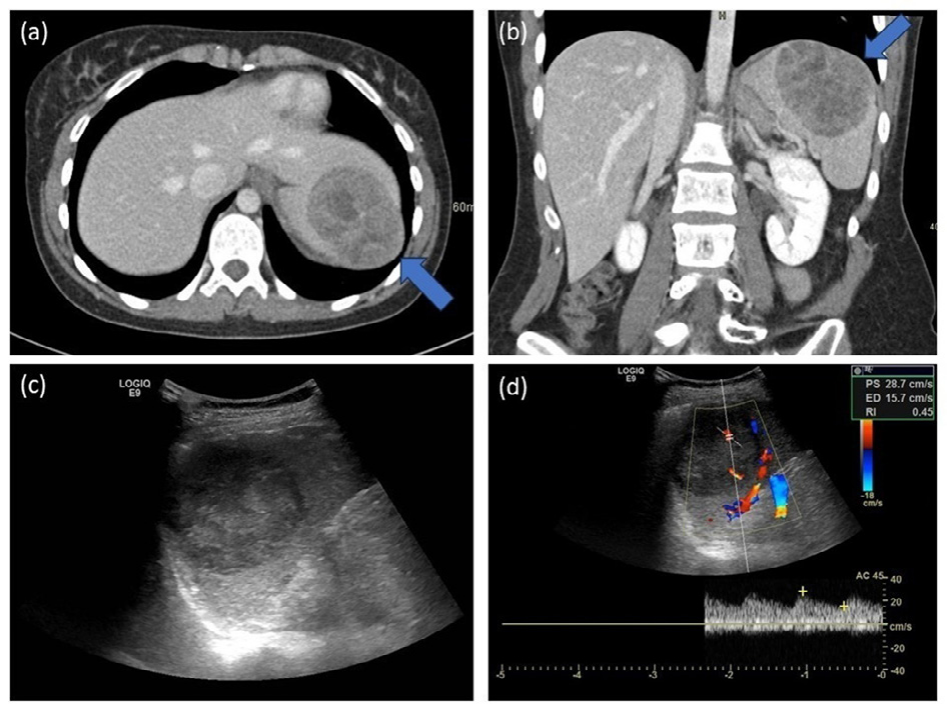
Splenic metastasis. **(a, b)** Abdominal CT. A sharply defined mass (5.5 × 7.2 × 5.8 cm), globally hypodense and inhomogeneously contrast-enhancing, located cranially and subcapsular in the spleen. Notice the bulging of the diaphragm (arrows), without signs of pleural/pulmonary invasion. **(c, d)** Abdominal US. A sharply defined, heterogeneous mass (5.5 × 7.2 cm) located cranially and subcapsular in the spleen. Colour Doppler shows present yet not pronounced vascularization.

Seven years later, however, an incidental splenic mass was discovered on an abdominal CT scan for recurrent urinary infections. On CT, the lesion presented as a heterogeneous hypodense and inhomogeneously contrast-enhancing lesion (Figure 2a and 2b). Sonography showed a well-defined heterogenic hypo-echogenic vascularized ovoid mass (Figure 2c and 2d). On PET-CT, the lesion was clearly hypermetabolic, and a malignancy could not be ruled out.

**Figure 3:**
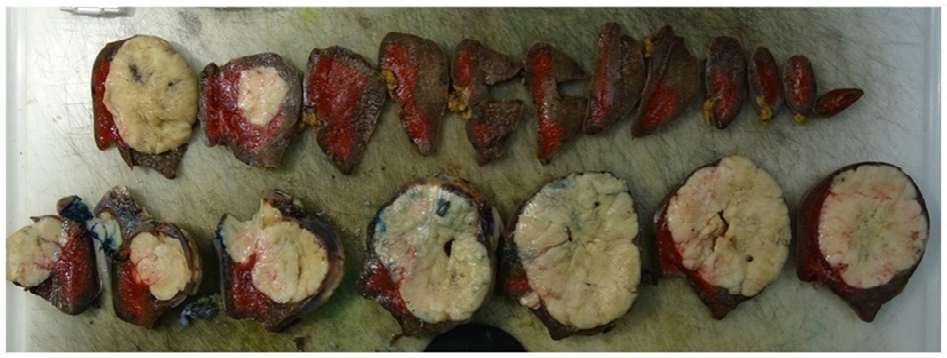
Macroscopic pathological examination of the spleen.

**Figure 4:**
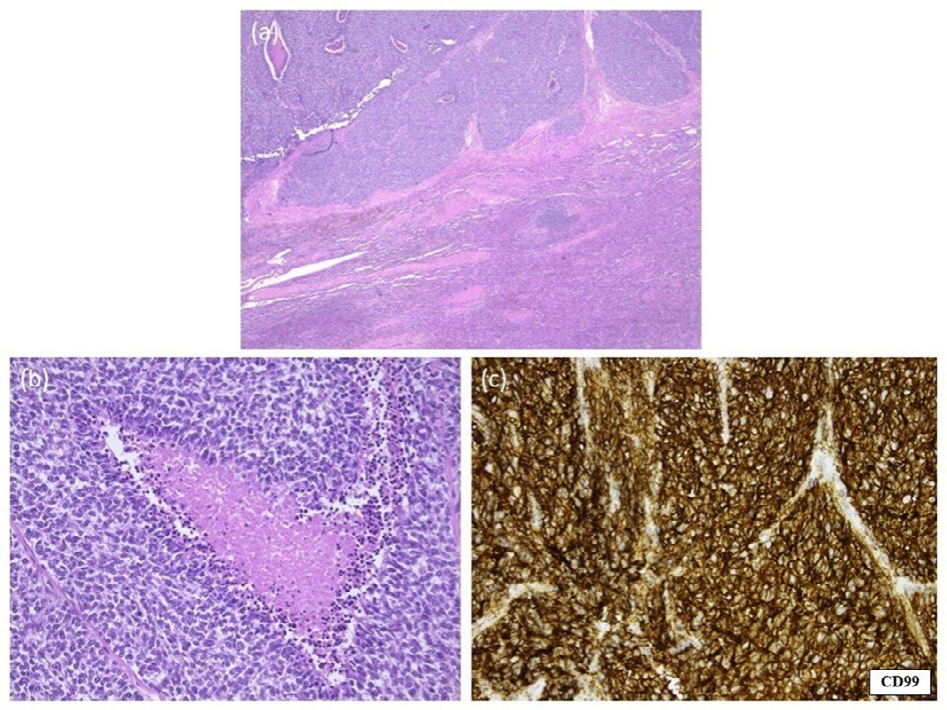
Microscopic pathological examination of the spleen. **(a, b)** Haematoxylin and eosin staining of a splenectomy specimen. The splenic tissue is diffusely invaded by small round blue cells and necrosis. Notice the fibrotic islands with surrounding characteristic cells. **(c)** Immunohistochemical staining of the splenectomy specimen. Strong, diffuse positivity for CD99 in a honeycomb pattern, correlating with high membranous expression typical for Ewing sarcoma.

A splenectomy was performed, and pathological examination (including typical CD99-positivity on immunohistochemistry and FISH-analysis) confirmed a splenic metastasis of the known lumbar ES (Figures 3 and 4). Treatment consisted of adjuvant chemotherapy. Two years later, without local recurrence or any new metastatic disease, the patient developed pleural metastases. She refrained from palliative chemotherapy and died five months later.

## Discussion

To our knowledge, metastatic involvement of the spleen in ES has not been described previously. Disseminated disease occurs in 20–25% of patients, with metastatic sites including mainly the lung (70–80%) and bone (40–45%) [8, 9]. Rare, atypical metastatic patterns like involvement of the small bowel, pancreas, oral and orbital cavities have been reported [1]. Atypical patterns of disseminated disease—especially extrapulmonary involvement—further worsen the survival rate [2].

Metastatic spread towards the spleen is rare in all types of malignancy and is present in only 2–9% of patients with end-stage malignant disease, although the spleen is a markedly vascularized organ [6]. It is hypothesized that the combination of biological and physical properties (spontaneous rhythmic contractility squeezing tumour emboli out, anti-neoplastic activity of lymphoid tissue, and the lack of an afferent lymphatic system) protects the spleen against nestling of metastases [10]. A splenic metastasis clinically often presents as a splenic rupture, demanding splenectomy.

Splenic lesions have a broad differential diagnosis: infectious and inflammatory processes, primary vascular or lymphoid neoplasms, vascular/ischaemic and systemic diseases, and rarely metastases [6, 7, 10]. Examples of solitary lesions are haemangioma, lymphoma, cystic masses, hamartoma, solitary fibrous tumour (SFT), inflammatory myofibroblastic tumour (IMFT), sclerosing angiomatoid nodular transformation (SANT) and angiosarcoma. When multiple lesions are present, inflammatory (sarcoidosis) and infectious (micro-abscesses) diseases, lymphangioma, littoral cell angioma and metastases should be considered.

## Conclusion

Ewing sarcoma is an aggressive malignancy that can present with uncommon patterns of disease dissemination. This case underscores the need for vigilance and the importance of maintaining a broad differential diagnosis when evaluating new splenic lesions or other parenchymal masses in patients with a history of oncological disease, even after long disease-free intervals. Recognition of such atypical presentations is crucial, as the detection of extrapulmonary metastases has significant prognostic and therapeutic implications. Radiologists play a key role in identifying these uncommon manifestations and guiding appropriate further management.
